# Molecular Characterization of Avulaviruses Isolated from Mallard Ducks in Moscow in 2008–2024

**DOI:** 10.3390/vetsci13010023

**Published:** 2025-12-25

**Authors:** Anastasia Treshchalina, Elizaveta Boravleva, Daria Gordeeva, Alexandra Gambaryan

**Affiliations:** Chumakov Federal Scientific Center for the Research and Development of Immune-and-Biological Products, Village of Institute of Poliomyelitis, Settlement “Moskovskiy”, 108819 Moscow, Russia; elisavetbor@gmail.com (E.B.); gordeeva.d.r@yandex.ru (D.G.); al.gambaryan@gmail.com (A.G.)

**Keywords:** Newcastle disease, avulavirus, NDV, APMV-4, wild waterfowl, migration

## Abstract

Species of the orders Charadriiformes and Anseriformes serve as the primary long-distance disseminators of various avulaviruses. The most economically significant among them is Newcastle disease virus (NDV), or Avian orthoavulavirus 1 (AOAV-1), which causes diseases of varying severity in both domestic and wild birds. Other avulaviruses have been studied to a much lesser extent, and for most of them, only single isolates are known, which does not allow a comprehensive assessment of their potential threat. To evaluate the biological diversity and potential risks posed by avian paramyxoviruses spread by wild waterfowl during autumn migration, fecal samples from mallards (Anas platyrhynchos) (*n* = 3604) were collected at water bodies in Moscow and the Moscow Region between 2008 and 2024. From these samples, AOAV-1 (*n* = 4) and Avian paraavulavirus 4 (APMV-4) (*n* = 9) were isolated and partially sequenced. Phylogenetic analysis revealed that all AOAV-1 isolates belong to genotype 1 of class II, while all APMV-4 isolates belong to the Eurasian subgenotype of genotype 1. Analysis of the F protein cleavage site motif indicated conformity with the consensus sequences characteristic of lentogenic and non-pathogenic avian paramyxoviruses in all isolates.

## Simple Summary

Waterfowl birds serve as reservoirs and vectors for a range of pathogens, including avulaviruses. The most well-known group of avulaviruses comprises Newcastle disease viruses, or Avian orthoavulavirus 1 (AOAV-1), which cause diseases of varying severity in more than 200 bird species. Between 2008 and 2024, 13 avulaviruses belonging to 2 species were isolated and partially sequenced from fecal samples of mallard ducks (Anas platyrhynchos). The isolated viruses belong to phylogenetic lineages widely distributed across Eurasia and are not highly pathogenic. The data obtained in this study reflect the genetic diversity of avulaviruses disseminated by mallards along migratory routes passing through Moscow.

## 1. Introduction

All avian paramyxoviruses, with the exception of turkey metapneumovirus, have been classified within the subfamily Avulavirinae of the family Paramyxoviridae [1]. Paramyxoviruses are enveloped, pleomorphic viruses, most commonly spherical in shape. On their surface, two glycoproteins are present: the fusion protein (F), which possesses fusogenic activity, and the hemagglutinin/neuraminidase protein (HN), which facilitates attachment by binding to sialic acid and also exhibits neuraminidase activity.

The subfamily Avulavirinae currently includes 28 species [2]. The greatest attention is focused on AOAV-1, also known as Orthoavulavirus javanense or Newcastle disease virus (NDV). This virus affects more than 200 bird species, and its detection on poultry farms requires the culling of entire flocks, resulting in significant economic losses. Although NDV and AOAV-1 are synonymous, not every strain causes clinical signs of Newcastle disease (ND). Depending on their pathogenicity in chickens, AOAV-1 strains are classified into velogenic, mesogenic, lentogenic, and nonpathogenic pathotypes. Waterfowl and shorebirds are considered the main reservoirs and disseminators of lentogenic NDV. In these hosts, the viruses generally do not cause severe symptoms; however, upon introduction into domestic poultry, the virus may begin evolving toward increased pathogenicity [3,4]. Phylogenetically, AOAV-1 based on the F protein gene is divided into two classes, I and II. Class I exhibits low evolutionary diversity, and all representatives are lentogenic viruses of wild birds except for a single strain [5]. Class II includes viruses with varying pathogenicity, infecting a wide range of bird hosts, and is further divided into at least 21 genotypes [6].

The study of other avulaviruses began relatively recently, and for most of them, only single isolates are known, making it difficult to investigate the genetic and phenotypic characteristics of these viruses and assess their potential risks. The first representatives of avulaviruses types 2–9 were isolated in the 1970s [7]. The subsequent expansion in the number of avulavirus groups has been associated with increased interest in wild bird influenza viruses, as the H5 influenza epizootic has led to the widespread implementation of wild bird influenza monitoring, with the unintended result of detecting a large number of previously unknown avulaviruses.

Moscow is located at the intersection of three major bird migration routes, and during autumn migration, a large number of birds pass through its urban water bodies, many of which do not come into contact during other seasons. In this study, we present the genetic and molecular analysis of avulaviruses isolated from mallards (Anas platyrhynchos) along the shores of water bodies in Moscow during the period 2008–2024.

## 2. Materials and Methods

### 2.1. Viruses

**Figure 1:**
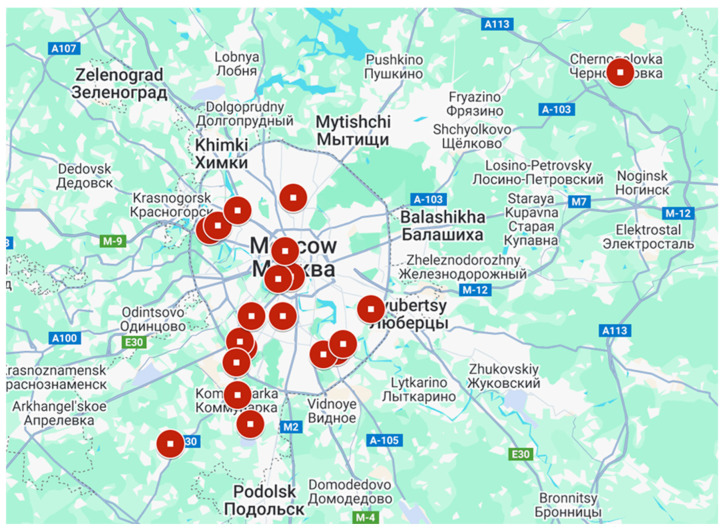
Places of collecting feces samples. The dots indicate sample collection locations: Troparevsky pond; Bolshoi Voroninsky pond; Cheremushkinsky pond; Udaltsovskie ponds; Vatutinki; Moscow river, Serebryanyy bor; Borisovskiye ponds; Verkhniy kuz’minskiy pond; Pionerskiy pond; park pond, Trubetskoy estate; Patriarshie Ponds; Bolshoy Stroginsky Zaton; Ivankovskie ponds, Pokrovskoye-Streshnevo park; Sredniy pond, Mosrentgen; Shkolnyi pond, Kommunarka; Butovskiy pond; Tsaritsyno ponds, Tsaritsyno Natural and Historical Park; Moscow river, Brateevo; Lake Yuzhnoye, Chernogolovka.

Fresh feces of healthy mallards were collected in 2008–2024 during August–November on the shore of city ponds in Moscow and Moscow region (Figure 1).

Feces were suspended in phosphate-buffered saline (PBS) supplemented with 2% MycoKill AB solution, 0.1 mg/mL kanamycin, 0.4 mg/mL gentamicin and 0.01 mg/mL nystatin. The suspension was centrifuged and 0.2 mL of the supernatant were inoculated into 10-day-old chicken embryos (CE). Allantoic fluid was collected after 48 h and tested by hemagglutination assay with chicken erythrocytes. Positive samples were taken for further passaging.

### 2.2. Sequencing

The viral RNA was extracted from infected allantoic fluid using QIAamp Viral RNA Mini Kit (QIAGEN, Hilden, Germany) following the manufacturer’s instructions. The reverse transcription reaction was carried out using the MMLV RT kit (Evrogen, Moscow, Russia) in the presence of a random decanucleotide primer. Polymerase chain reaction was carried out with the Tersus Plus PCR Kit (Evrogen, Moscow, Russia). Oligonucleotides used in the work: fFapmv2-(ATGGGCTCCAGACCTTCTAC); rFapmv2-(CTGCCACTGCTAGTTGCGATAATCC); fFapmv4(v2) (CAAYCAYAATGAGGYTATCAMRCACAATC); rFapmv4 (CTAGAAAGGCGTCCCYAATTTAGTGG). These oligonucleotides are targeted to conserved regions of the genome and were selected using the SnapGene Viewer program (https://www.snapgene.com/snapgene-viewer (accessed on 13 February 2025)) and the OligoAnalyzer Tool online service (https://eu.idtdna.com/pages/tools/oligoanalyzer?returnurl=%2Fcalc%2Fanalyzer, accessed on 13 February 2025). Analysis of the PCR results was performed by electrophoresis in a 1.5% agarose gel in Tris-acetate buffer. PCR fragments were excised for gel purification with the Qiagen MinElute Gel Extraction Kit (QIAGEN, Hilden, Germany) according to the manufacturer’s instructions. The nucleotide sequence of the gene fragments was obtained by Sanger sequencing on an ABI PRISM 3130 Genetic Analyzer (Applied Biosystems, ThermoFisher Scientific, Waltham, MA, USA) using an ABI PRISM^®^ BigDye™ Terminator v. 3.1 (ThermoFisher Scientific, Waltham, MA, USA).

### 2.3. Phylogenetic Analysis

Sequence processing was performed using BioEdit 7.2. and MEGA 12 (https://bioedit.software.informer.com/, accessed on 3 October 2022) and (https://www.megasoftware.net/, accessed on 3 March 2025). For AOAV-1, several sequences from representative strains of each class II genotype were taken for the initial sample [6]. After the preliminary samples were compiled, nucleotides were added to the sequence of the Moscow isolates and a repeat of the 10 most homologous relatives according to BLAST 2.17.0 for each, after which the sample was checked for presence. All participants in the database took part in the selection of APMV-4 according to their numbers. All nucleotide sequences were aligned using the MUSCULE algorithm and cut in the reading frame. Maximum-likelihood trees based on a K2P+G+I model (OAOV-1) and T92+G+I model (APMV-4) with 1000 bootstrap replicates were constructed by using the MEGA 12 [8]. The iTOL v7.2 online service (https://itol.embl.de/, accessed on 12 May 2025) was used to visualize and annotate the tree. Genotype identification was carried out on the basis of phylogenetic topology.

## 3. Results

Between 2008 and 2024, a total of 3604 fresh mallard fecal were collected during the autumn bird migration season along the shores of water bodies in Moscow and the Moscow region. Using RT-PCR, genetic material of AOAV-1 was detected in four samples, and APMV-4 in nine samples. All avulaviruses were isolated from samples collected at Troparevo Pond (south-west Moscow) in different years. Samples collected at the remaining 18 sites tested negative for avulaviruses but contained avian influenza viruses [9].

The primary structures of F gene fragments, including the region encoding the F0 cleavage site, were determined, and the obtained nucleotide sequences were deposited in the GenBank database (https://www.ncbi.nlm.nih.gov/genbank/, accessed on 13 February 2025) (Table 1).

**Figure 2:**
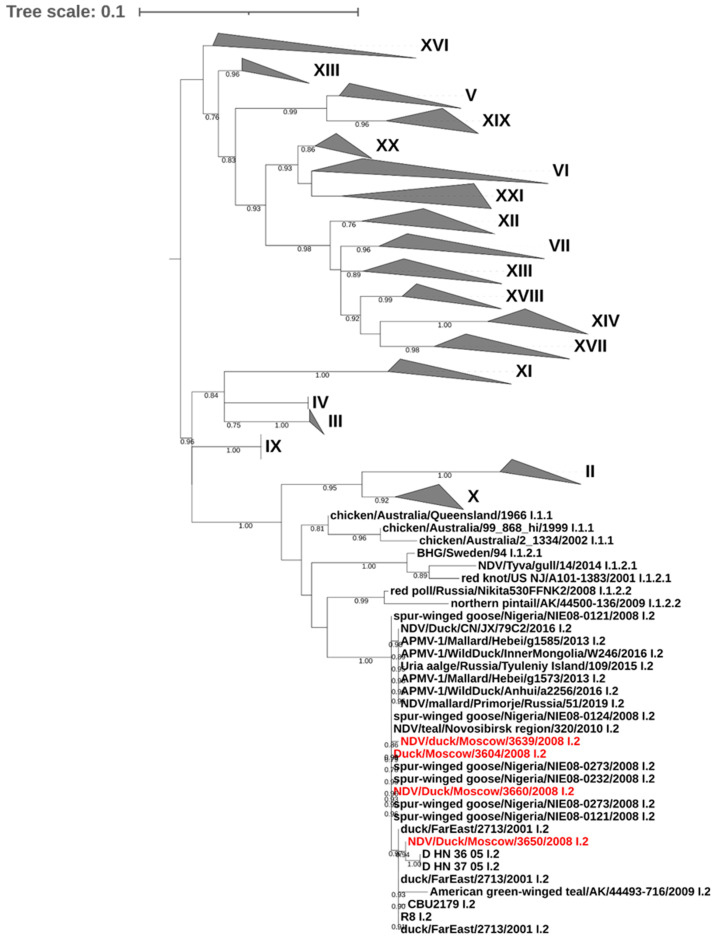
AOAV-1 phylogenetic tree. Maximum likelihood tree includes 173 nucleotide sequences with 301 positions. Viruses sequenced in Moscow as part of this study are highlighted in red. Nodes indicate bootstrap support of maximum likelihood ≥ 0.75. All genotypes except genotype VII are compressed. Subgenotypes of genotype VII are shown next to the virus names.

Phylogenetic analysis of the F gene fragment demonstrated that all four AOAV-1 isolates from mallards in Moscow belonged to class II, genotype I, subgenotype 2 (Figure 2). This genotype is distributed throughout the Eastern Hemisphere and has a wide range of avian hosts. The analyzed isolates formed a single cluster comprising a mixture of viruses of European, Asian, and African origin, isolated from wild aquatic and waterfowl birds. Among the closest relatives were viruses from China, isolated in 2005 (FJ597618, FJ597619), and viruses from Nigeria, isolated in 2008 (HG326605, HG326607, HG326608).

The first strain of Paraavulavirus hongkongense, or APMV-4, was isolated in 1975 and was rarely detected for a long period thereafter. As recently as 2012, the entire sequence diversity in GenBank was represented by two “reference” viruses: APMV-4/duck/BE/15129/07 from Belgium and APMV-4/duck/HK/D3/75 from Hong Kong, as well as a small group of viruses from South Korea [10]. Although the database of sequenced viruses has gradually expanded, the number of complete sequences remains very limited. Currently, all APMV-4 isolates are classified into four genotypes and a separate branch containing APMV-4/duck/HK/D3/75 based on phylogenetic analysis [11].

**Figure 3:**
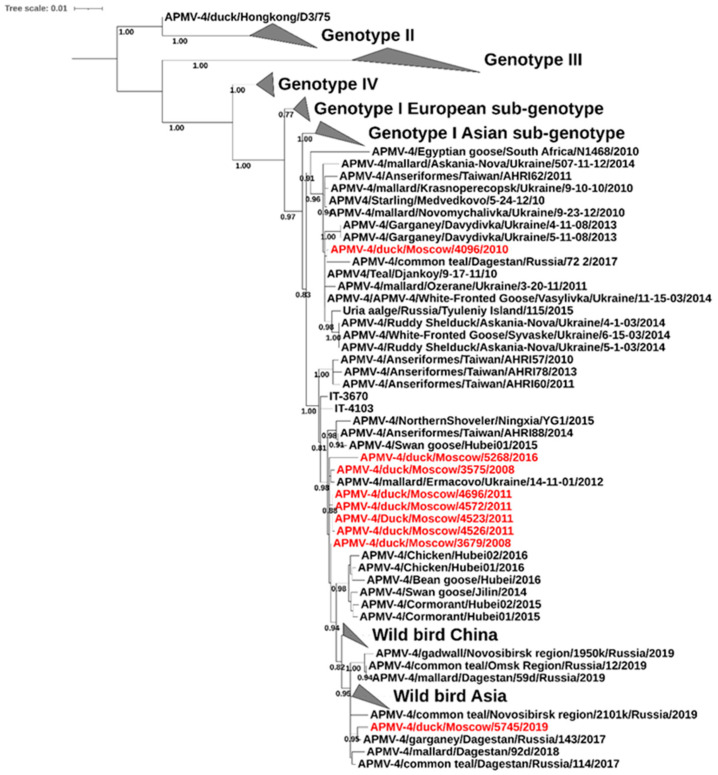
Phylogenetic tree of APMV-4. Maximum likelihood tree includes 180 nucleotide sequences with 1627 positions. Viruses from Moscow sequenced in this study are highlighted in red. Nodes indicate bootstrap support of maximum likelihood ≥ 0.75. All genotypes except Eurasian subgenotype of genotype I are compressed.

All analyzed isolates from Moscow belonged to the Eurasian subgenotype of genotype I, which also includes isolates from Russia, Ukraine, China, Italy, and countries on the African continent. The phylogenetic tree (Figure 3) shows that the Moscow isolates split into two clades within the subgenotype. The isolate APMV-4/duck/Moscow/4096/2010 clustered predominantly with Ukrainian viruses, while the remaining isolates grouped within a larger cluster of primarily Asian viruses. Within this Asian cluster, the Moscow isolates further divided into two groups, suggesting at least three independent APMV-4 introduction events into the region. The closest phylogenetic relatives of the Moscow isolates were viruses from the Republic of Dagestan (Russia) (PP537556, PP537559) and Ukraine (KT732340).

Although the ICPI test is typically used for definitive pathogenicity assessment of AOAV-1, the WOAH considers it impractical for isolates obtained from healthy birds during surveillance and recommends analysis of the F0 cleavage site as an alternative [12]. Analysis of the amino acid sequences of the AOAV-1 F protein revealed monobasic cleavage sites in the isolates: three isolates with ^109^SGGGKQGRLIG^119^ and one isolate with ^109^SGGEKQGRLIG^119^. The cleavage site in all APMV-4 isolates was also examined and found to be monobasic, represented by ^116^DIQPRF^121^. Thus, the amino acid sequences of the cleavage sites in the isolates obtained during this study fully or partially correspond to the consensus sequences typical for lentogenic and non-pathogenic avian paramyxoviruses [13].

## 4. Discussion

Currently, laboratories around the world are engaged in the isolation and study of avian viruses. However, the majority of studies remain focused on NDV, while other avulaviruses continue to be understudied. Research on wild bird viruses is often highly localized, resulting in the absence of data on avian virus circulation in certain regions in public databases, which prevents a comprehensive assessment of the overall situation.

We isolated and partially sequenced all viruses that yielded positive results in the hemagglutination assay and were subsequently confirmed by RT-PCR. The percentage of avulavirus-positive samples was 0.43% of the total number of samples, which is generally consistent with the isolation frequencies reported by other authors. Most of the avulaviruses in this study were isolated from samples collected between 2008 and 2011, with only two isolates obtained in subsequent years. A similar pattern was observed for influenza viruses [14]. One possible cause could be climate change. It has been shown that each year, more and more birds remain in the city for the winter, which reduces the spread of the virus [14,15]. It also remains unclear why avulaviruses were detected only in a single pond, while influenza viruses isolated from the same set of samples were recovered from a larger number of sites. Other studies frequently report coinfections of these two viruses, which was not observed in the present work [16,17].

Geographically, the isolates from Moscow are related to viruses from Southeast Asia, Eastern Europe, and the Middle East. The wide range of geographic origins of related viruses suggests active, ongoing exchange of viruses across distant regions throughout the Eastern Hemisphere. In this study, four AOAV-1 isolates belonging to subgenotype I.2 of class II were identified. According to previous reports, representatives of this group have also been isolated from wild birds in Yakutia, Dagestan, and the Amur and Vladimir regions of Russia [18,19,20].

APMV-4 is typically isolated from wild birds as a byproduct during avian influenza virus surveillance programs. Despite the large scale of avian influenza monitoring programs, the number of sequenced APMV-4 genomes in databases remains low, resulting in a limited understanding of this group of viruses [21,22]. In the present study, nine APMV-4 isolates were obtained. All studied isolates exhibited a monobasic F0 cleavage site, indicating their apathogenic or lentogenic pathotypes. Overall, according to current research findings, APMV-4 is not considered to have significant relevance for domestic poultry, as strains do not cause severe disease or pathology in chickens under laboratory conditions, and antibodies against them do not provide protection against subsequent AOAV-1 infection [23]. Furthermore, these viruses are considered to be of low pathogenicity for their primary wild bird hosts as well. These factors may reduce the perceived attractiveness of studying this group of viruses. Globally, most research has focused on AOAV-1 due to its economic importance [24]. However, the study of such viruses and the submission of related data to databases are essential for expanding our understanding of virus dissemination mechanisms among wild birds and inter-virus interactions. Continued monitoring of viruses in wild birds is also necessary for tracking virus transmission routes, which will aid in the timely prediction of potential introductions of dangerous viruses.

## Figures and Tables

**Table 1:** F gene fragments of Avulaviruses isolated in Moscow in 2008–2024.

№	Isolate	Subtype	Genbank Source Sequence Accession	Collection Date
1	APMV4/duck/Moscow/3575/2008	APMV-4	PV929733	18 October 2008
2	NDV/duck/Moscow/3604/2008	AOAV-1	PP780192	18 October 2008
3	NDV/duck/Moscow/3639/2008	AOAV-1	PP780193	10 August 2008
4	NDV/duck/Moscow/3650/2008	AOAV-1	PV929740	10 August 2008
5	NDV/duck/Moscow/3660/2008	AOAV-1	PV929741	21 October 2008
6	APMV4/duck/Moscow/3679/2008	APMV- 4	PV929734	21 October 2008
7	APMV4/duck/Moscow/4096/2010	APMV-4	PV929735	8 October 2010
8	APMV4/duck/Moscow/4523/2011	APMV-4	PV929736	4 October 2011
9	APMV4/duck/Moscow/4526/2011	APMV-4	PV929737	4 October 2011
10	APMV4/duck/Moscow/4572/2011	APMV-4	PV929738	4 October 2011
11	APMV4/duck/Moscow/4696/2011	APMV-4	PP756625	15 November 2011
12	APMV4/duck/Moscow/5268/2016	APMV-4	PP756624	25 October 2016
13	APMV4/duck/Moscow/5745/2019	APMV-4	PV929739	15 October 2019

## Data Availability

The original contributions presented in this study are included in the article. Further inquiries can be directed to the corresponding author.
